# The Second Skin of Seagrass Leaves: A Comparison of Microalgae Epiphytic Communities Between Two Different Species Across Two Seagrass Meadows in Lesser Sunda Islands

**DOI:** 10.21315/tlsr2021.32.2.7

**Published:** 2021-06-29

**Authors:** Putu Satya Pratama Atmaja, Dietriech G. Bengen, Hawis H. Madduppa

**Affiliations:** Department of Marine Science and Technology, Faculty of Fisheries and Marine Science, IPB University, Jl. Rasamala, IPB Darmaga Campus, Bogor 16680, Indonesia

**Keywords:** Biofilms, Microbiome, Seagrass, Biodiversity, Indonesia

## Abstract

Epiphytes as the important features in the seagrass ecosystems have been studied widely, and their functions as a primary producer, influence rates of herbivory grazer, and prevent seagrass leaf from desiccation is well known. However, patterns and distribution among seagrasses especially in Indonesia, which was known as hotspot marine biodiversity is not well understood. Therefore, this study aimed to examined epiphytic assemblages on two seagrass species with different morphological and longevity, *Enhalus acoroides* and *Cymodocea rotundata*, in two different meadows (conservation area and non-conservation area) in Lesser Sunda Islands (Bali and Lombok). A total of 22 taxa of microalgae epiphytes species were identified from eight sites and 2 different species of seagrass. The highest number of collected species between class was from Bacillariophyceae (18), followed by Cyanophyceae (3) and Fragilariophyceae (1). Analysis of similarity (ANOSIM) revealed a significant difference of microalgae epiphytes assemblages between sites and seagrasses. Epiphytes assemblages in conservation area were more abundant than non-conservation area, both in Bali and Lombok. On seagrass comparison, *Enhalus acoroides* showed higher abundance of epiphytes assemblages than those on *Cymodocea rotundata*. Based on principal component analysis (PCA), this study highlights the microalgae epiphytic communities strongly influenced by seawater temperature, phosphate’s concentration, and pH in sediment. This study also demonstrated that the assemblages of microalgae epiphytic communities affected by differences of seagrass morphological and longevity.

**Highlights**

- Analysis of similarity (ANOSIM) revealed a significant difference of microalgae epiphytes assemblages was found between sites (conservation area and non-conservation area) and seagrasses (*Enhalus acoroides* - *Cymodocea rotundatta*).
- The assemblages of microalgae epiphytes was strongly influenced by seawater temperature, phospate and pH in sediment.
- The differences of microalgae epiphytes assemblages in the seagrass leaf was driven by differences of seagrass morphological and longevity.

## INTRODUCTION

The seagrass is unique among flowering plants which have adapted to live immersed in seawater. They produce seeds, flower and pollinate completely underwater. Seagrass also rank with coral reefs and mangroves as some of the world’s most productive and ecologically significant marine ecosystems (Shaffai 2011). The seagrass leaves provide suitable substrate for the establishment and growth of a number of epiphytes which form laminates assemblages characterised by high species diversity (Kocak & Aydin-Onen 2014; Leliaert *et al*. 2001). The epiphytic communities is a natural part of seagrass leaves that may contributes significantly to the overall seagrass productivity and contains lots of important microorganism such as diatoms (Bacillariophyceae), cyanobacteria, and bryozoans species (Chung & Lee 2008; Kocak & Aydin-Onen 2014; Uku *et al*. 2007). Additionally, epiphytes provide support to higher tropic and directly influence rates of herbivores grazer (Marco-Méndez *et al*. 2015; Prado *et al*. 2007). The epiphytes presence also prevent the leaves of seagrass from desiccation because of UV radiation, so that increasing 67% growth of seagrass (Aho & Beck 2011). However, high anthropogenic activities can directly affect the nutrient level in sea water that may influence the epiphytes load. In fact, high levels of nutrients have been associated with increasing the density of epiphytes (Nelson 2017). As the result, high dense of epiphytic cover leads the photosynthetic activities of seagrass decreased and reduce survival rates because of light limitation (Orbita & Mukai 2013).

Aside of light limitation and nutrients, there are several factors that influenced epiphytic community structure such as water motion, grazing pressure by herbivores, leaf length and leaf age (Lavery *et al*. 2007; Mabrouk *et al*. 2014). The oldest leaf often colonised by higher diversity (68%–92% of the epiphytes biomass per shoot) than youngest leaf (Reyes & Sanson 2001). This is strongly related to the total leaf surface that available to the epiphytes to settle on and grow. Seagrass with distinct morphological differentiation tend to provide several distinct microhabitats for epiphytes. Shift in numerical dominance between certain species occurred in fall, spring and summer (Medlin & Juggins 2018). This shifted colonisation, abundances, and the functional of dominances suggested competition for substrate surface area. Structure of epiphytes communities is similar to higher plant succession in the consistent change in vertical community structure from low to high physical stature, in the association of numerical dominance with large stature (via cell size or long mucilaginous stalks), and in the progressive slowdown in the rate of succession.

Studies about epiphytic communities have been widely carried out (Crump & Koch 2008; Güreşen *et al*. 2020; Hamisi *et al*. 2013; Lobelle *et al*. 2013). Although a good number of studies focused on epiphytes in sub-trophic and Mediterranean area, Indonesia especially Bali and Lombok have received little attention. The purpose of this study is to investigate the community structure of microalgae epiphytic on *Enhalus acoroides* and *Cymodocea rotundata* leaves in two different meadows, which were in non-conservation area and conservation area at Bali and Lombok. This study aimed to examine the following hypothesis: (1) due to their larger leaves area, *E. acoroides* may have more abundant and more diverse epiphytic microalgae than those on *C. rotundata*; (2) Community structure of epiphytic microalgae in conservation area is more diverse than non-conservation area; (3) Variation of sea water parameters and sediments may play an important role in term of epiphytic microalgae pattern.

## MATERIAL AND METHODS

### Study Sites

**Figure 1:**
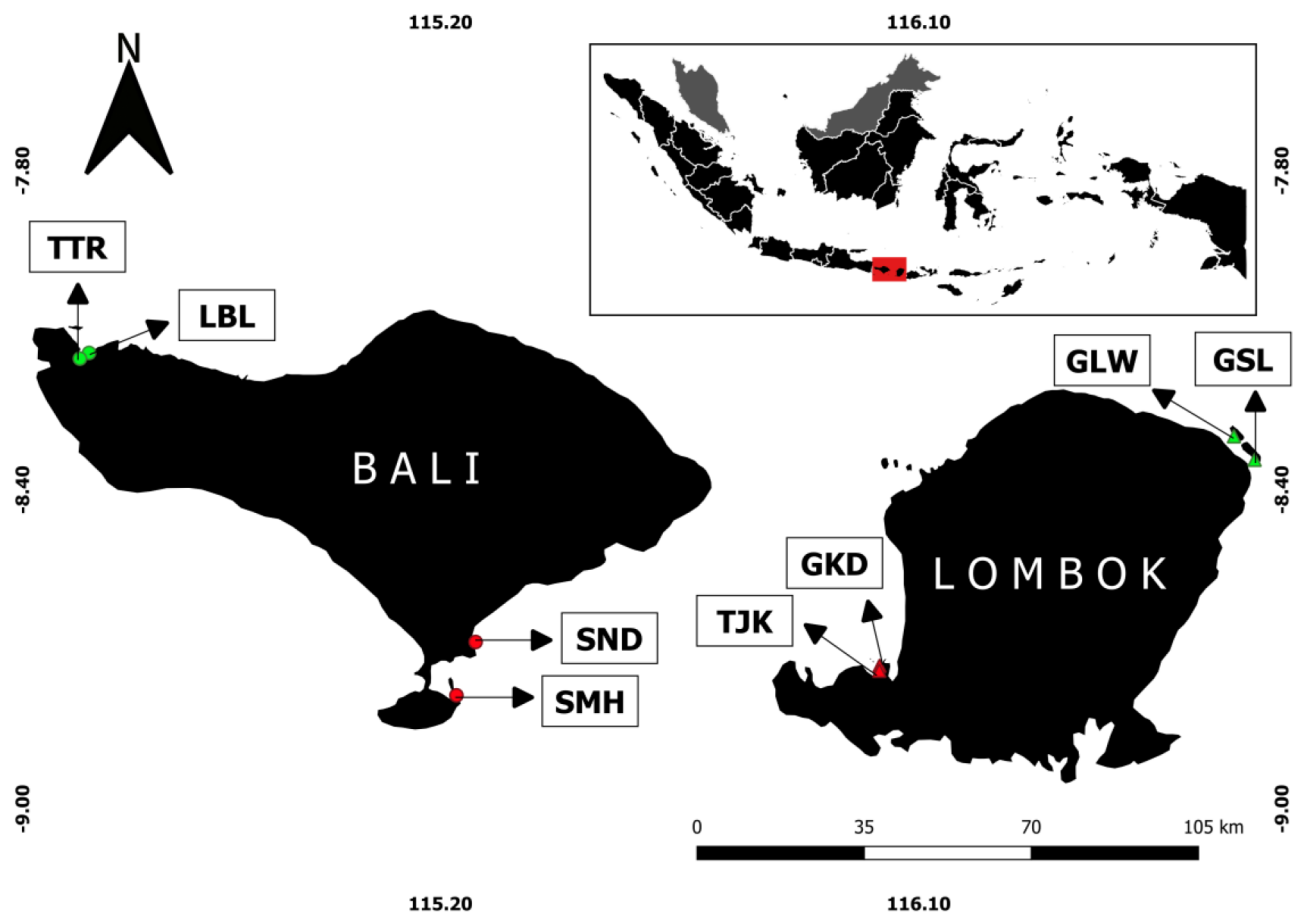
Location map of *Enhalus acoroides* and *Cymodocea rotundata* sampled from Bali and Lombok: refer sites name and codes from Table 1. (*Source*: Google Map and re-visualise using QGIS).

This study was conducted in two islands, Bali and Lombok. Each island consists of two sites which represent non-conservation area (NCA) and conservation area (CA) (Table 1, Fig. 1). There were two main attributes that distinguish NCA and CA. The first one lies in protection of each area. The conservation area exists to manage and protect the certain species or special architecture. A specific permit was needed to enter or access the area. The contribution of conservation area is to ensure the sustainability and strengthen connectivity between populations, assemblages, and ecosystem functions in seascapes (Olds *et al*. 2016). Secondly, it was about the possible disturbance of high anthropogenic pressure, which can directly affect the marine ecosystems and their functions (Duarte 2000). The possible anthropogenic pressure is higher in NCA than in CA because the protection of NCA was lower than CA.

Bali was conducted in Samuh (SMH) and Shindu (SND) as represent for NCA and in Teluk Terima (TTR) and Labuhan Lalang (LBL) for CA. Lombok was conducted in Tanjung Kelor (TJK) and Gili Kedis (GKD) to represent NCA and in Gili Lawang (GLW) and Gili Sulat (GSL) as representative for CA. These areas were selected based on conservation status and presence of two seagrass species target, which was *E. acoroides* and *C. rotundata*.

### Data Collection

Data collection were conducted in dry season during expeditions in October 2019 in Bali and November 2019 in Lombok. All samples were collected in the morning at low tide condition to makes counting shoot density possible. Shoots were collected using a metal square quadrat (0.25 m^2^). The quadrat was randomly placed over shoots, which seagrass species target found (*E. acoroides* and *C. rotundata*). At each station, five replicates quadrats were sampled. The shoot density of *E. acoroides* and *C. rotundata* was counted and expressed as number of shoots/m^2^. After shoots was counted, at each station, two shoots of *E. acoroides* and *C. rotundata* were collected. A total of 10 *E. acoroides* and *C. rotundata* leaves was sampled from each site. The amount of shoots taken in this study was based on Global Seagrass Research Methods (Short & Coles 2001). For each shoot, length of oldest leaf and leaf width were scored. Leaf area index (LAI) was determined as product of leaf surface area (total leaf length x mean leaf width) and expressed as m^2^/shoot (Mabrouk *et al*. 2014).

After all the shoots were counted, microalgae epiphytes was scratched gently using clean and steril razor blade 10 cm from tip of the oldest leaves, to ensure that this study focused on a high dense and mature assemblages of epiphytes colonisation (Orbita & Mukai 2013; Pardi *et al*. 2006). This procedure was applied to all shoots from both targeted seagrass leaves and preserved in 100 mL bottle contained of 4% formalin, seawater and lugol (Piazzi *et al*. 2016). In the laboratory, all bottles contained of microalgae epiphytes material were investigate and examined (three repetition) under the microscope using Sedgwick Rafter Counting Cell. All the microalgae epiphytes was identified based on their morphology to lowest possible taxon using identification book entitled Coastal Plankton Photo Guide for European Seas (Larink & Westhide 2006) and Marine Phytoplankton: Selected Microphytoplankton Species from the North Sea Around Helgoland and Sylt (Hoppenrath *et al*. 2009).

Physicochemical parameter and sediments also measured during the study from each site on both islands (Table 2). There were six seawater parameters taken from this study, which were temperature, salinity, dissolved oxygen, pH, nitrate and phosphate. Meanwhile sediment parameters were pH, eH and texture analysed in laboratory.

#### Data analysis

The community indices in this study consisted of three components which were the Shannon-Wiener index (H′), Evenness index or similarity index (E), and dominancy index (C). The Shannon-Wiener index (H′) was used to analyse how many different species of microalgae epiphytes were found in a community (Odum 1971). The Shannon-Wiener index was calculated using the following formula:

$$H^{\prime }=-\sum_{i=1}^{n}{\text{p}}_{i}\text{ln}p_{i}$$

where,

*n* = number of species
*p_i_* = proportion of individuals
*ln* = log base *e*

Evenness index or similarity index (E) was used to analyse the similarity or flatness of species from epiphytes community. The Evenness index (E) was calculated using the following formula:

$$E=\frac{H^{\prime }}{\text{ln}S}$$

where,

*H′* = the Shannon-Wiener index
*S* = amount of taxa counted
*ln* = log base *e*

While the dominancy index (C) was used to analyse the species, which were most commonly found and dominate in a community. The dominancy index (C) was calculated using formula:

$$C=\sum_{i=11}^{n}p_{i}^{2}$$

where,

*n* = number of species
*p_i_* = proportion of individuals

Analyses of similarity (ANOSIM) randomisation tests (with untransformed data) were used to test for differences in species abundance among sampling stations and good for heterogeneity in dispersion (Short & Coles 2001; Anderson & Walsh 2013). If differences were found using ANOSIM, then similarity percentages – species contribution (SIMPER) analysis was used for identifying which species primarily accounted for observed differences in epiphytic assemblages between sampling stations. SIMPER generates a ranking of the percent contribution of species that are most important to the significant differences. SIMPER used to know the magnitude of the contribution of certain species to their spread based on their similarity (Clarke 1993).

Non-metric multidimensional scaling (nMDS) was used to analyse the patterns distribution of microalgae epiphytes on both leaves. Unlike PCA, nMDS depended on sequence and distance. Also it was used to represent the original position of the data in a multidimensional space as accurately as possible using a reduction in the number of dimensions, so it would be able to be plotted and visualised (Cheng 2004). The ANOSIM, SIMPER and nMDS statistical analysis was conducted using PAST 4.0.

The relations between epiphytic communities and physicochemical parameters in seawater and sediment was enhanced through Principal Component Analysis (PCA). All the parameters that taken in this study was selected as independent variables for the PCA, except texture because it was non-numerical data. The PCA analysis was conducted using STATISTICA 13.0.

## RESULTS

### Density and Leaf Area Index (LAI) of Seagrass

*C. rotundata* exhibits the highest shoot density while *E. acoroides* has the highest LAI. *C. rotundata* in Lombok was denser than in Bali, while for *E. acoroides* in Bali was denser than in Lombok. The average of density was 86.46 ± 9.04 shoot/m^2^ and 45.01 ± 5.95 shoot/m^2^ for *C*. *rotundata* in Lombok and Bali, respectively. Meanwhile the average density of *E. acoroides* in Bali was 32.93 ± 4.68 shoot/m^2^ and 30.59 ± 10.02 shoot/m^2^ for Lombok. LAI for *E. acoroides* was 10.157 ± 0.95 m^2^/shoot and 6.574 ± 3.37 m^2^/shoot in Bali and Lombok, respectively. Whilst, LAI for *C*. *rotundata* in Bali was 0.589 ± 0.08 m^2^/shoot and 0.491 ± 0.1 m^2^/shoot in Lombok. The highest mean value of leaf length (86 ± 7.07 cm) was recorded for *E. acoroides*, whereas the lowest mean value (4.9 ± 1.14 cm) was observed for *C. rotundata*.

### Species Richness and Abundance

A total of 22 taxa of microalgae species were identified from eight sites and two different species of seagrass. The highest number of collected species was from Bacillariophyceae (*diatoms*) class (18), followed by Cyanophyceae (3) and Fragilariophyceae (1). Tables 3 and 4 showed species richness comparison of microalgae epiphytes between Bali and Lombok on *Enhalus acoroides* (EA) and *Cymodocea rotundata* (CR). Conservation area in Bali which was consist of two sites, Teluk Terima (TTR) and Labuhan Lalang (LBL) exhibits higher assemblages of microalgae epiphytes than Samuh (SMH) and Shindu (SND) which represent non-conservation area. The total taxa of epiphytes species on *E. acoroides* (22) was observed higher than *C. rotundata* (18). The highest species recorded was *Nitzchia sp*. (163 ± 22.61), whereas *Navicula sp*. (1 ± 1.37) observed for the lowest species. The most frequent species was *Nitzchia sp*. (163 ± 22.61) and (95.6 ± 28.85) for *E. acoroides* and *C. rotundata*, respectively. Meanwhile the lowest species counted was (0.87 ± 1.93) for each *Biddulphia sp*. and *Fragillaria sp*. on *Enhalus acoroides*, whereas *Navicula sp*. (1 ± 1.37) and *Gyrozigma sp*. (1 ± 2.23) counted as the lowest species found in *C. rotundata*.

In contrast, Table 4 showed that NCA (13) in Lombok which was consist of Tanjung Kelor (TJK) and Gili Kedis (GKD) had higher amount of taxa than CA (11). Meanwhile on seagrass comparison, the total taxa of epiphytes species in *E. acoroides* (16) was higher than *C. rotundata* (10). *Nitzchia* sp. (140 ± 10.7) was recorded as the most frequent species found, whereas *Lyngbya* sp. (1.53 ± 1.57) was counted as the lowest species. The most frequent species found was *Nitzchia* sp. (140 ± 10.7) and *Oscillatoria* sp. (44.2 ± 10.75) for *E. acoroides* and *C. rotundata*, respectively. Whilst the lowest species counted on *E. acoroides* was *Coscinnodiscus* sp. (1.33 ± 2.34) and *Lyngbya* sp. (1.53 ± 1.57) for *C. rotundata*.

The average of epiphytes abundance in conservation area in both locations were higher than non-conservation area. The most abundant species was *Nitzchia* sp. (2.68 ± 0.97 ind/mm^2^), while the lowest was *Biddulphia* sp. (0.008 ± 0.006 ind/mm^2^) and *Fragillaria* sp. (0.008 ± 0.006 ind/mm^2^). Abundance of those species were included in Bacillariophyceae group.

*E. acoroides* had higher abundant of microalgae epiphytes than *C. rotundata*. *Nitzchia* sp. (2.68 ± 0.97 ind/mm^2^) was the most abundant species counted on *E. acoroides* leaves, while the lowest species was *Biddulphia* sp. (0.008 ± 0.006 ind/mm^2^) and *Fragillaria* sp. (0.008 ± 0.006 ind/mm^2^). *Nitzchia* sp. (1.3 ± 0.42 ind/mm^2^) also observed as the most abundant species on *C. rotundata*, while the lowest species was *Navicula* sp. (0.04 ± 0.08 ind/mm^2^).

Analysis of similarity (ANOSIM) in all test pairs showed the significant difference in diversity of microalgae epiphytes between sites and between seagrass in Bali and Lombok (Table 5).

### Community Structure of Microalgae Epiphytes

The community structures consist of Shannon-Wiener diversity index (H′), evenness index (E) and Simpson index (C) are shown in Table 6 and describe as follows. The diversity index (H′) among NCA and CA in Bali and Lombok were categorised as moderate. The evenness index (E) on all sites showed that the value closed to 1 and were categorised as high. The Simpson index (C) showed all the value closed to 0 in all sites and were categorised as low.

The same result also showed on community structure of *E. acoroides* (EA) and *C. rotundata* (CR). Diversity index (H′) between both seagrasses in Bali and Lombok were categorised as moderate. The evenness index (E) on all seagrasses were categorised as high because the value closed to 1. The Simpson index (C) showed all the value closed to 0 among the seagrasses and were categorised as low.

As significant difference was found, a similarity percentage (SIMPER) was applied (Table 7). Analysis of SIMPER showed that the average dissimilarity was high between sites (61.73%) and between seagrass (53.1%). The highest species that contributes dissimilarity between sites was *Nitzchia* sp. (24.52%), followed by *Lycmophora* sp. (15.93%), *Oscillatoria* sp. (12.96%), and the rest are less than 10%. Meanwhile the highest species contribution between *E. acoroides* and *C. rotundata* was *Lycmophora* sp. (26.82%), *Coscinnodiscus* sp. (11.74%), *Oscillatoria* sp. (11.52%), *Nitzchia* sp. (11.33%), and the rest are less than 10%.

### Physicochemical and Sediment Parameter

The variabilities of physicochemical in seawater and sediment showed in Table 8. In general, seawater temperature in Lombok was higher than in Bali. The highest temperature measured was 34 ± 1.09°C, while the lowest was 26.73 ± 0.6°C. The salinity concentration in Lombok also relatively higher than in Bali. The highest salinity was 35.67 ± 0.72‰, while the lowest was in Samuh (SMH) (28.87 ± 0.63‰).

However, the concentration of dissolved oxygen in Bali relatively higher than in Lombok. The highest measured of dissolved oxygen’s concentration was 7.52 ± 0.21 mg/L, while the lowest was 3.72 ± 0.24 mg/L. The pH concentration in all sites at Bali and Lombok were relatively same each other, with the average concentration of 7.67 ± 0.17 for Bali and 7.68 ± 0.13 for Lombok. In general, the concentration of nitrate in Lombok was higher than in Bali, while in contrast the phospate’s concentration in Bali was higher than in Lombok.

Meanwhile in sediment where all the seagrass and epiphytes sample taken, the concentration of pH ranged from 6.8 to 7.4 with the average of 7.32 ± 0.09 for Bali and 6.92 ± 0.12 for Lombok. The concentration of redox potential on sediment in Bali was 0.5 higher than in Lombok. The highest concentration measured was 110.2 ± 17.16 mV, while the lowest was 48 ± 13.44 mV. All the texture of sediments were categorised as sand, except for Teluk Terima (TTR) and Labuhan Lalang (LBL) which were categorised different as sandy clay.

### Microalgae Epiphytic Distributions and Its Relationship to Physicochemical Parameters

**Figure 2:**
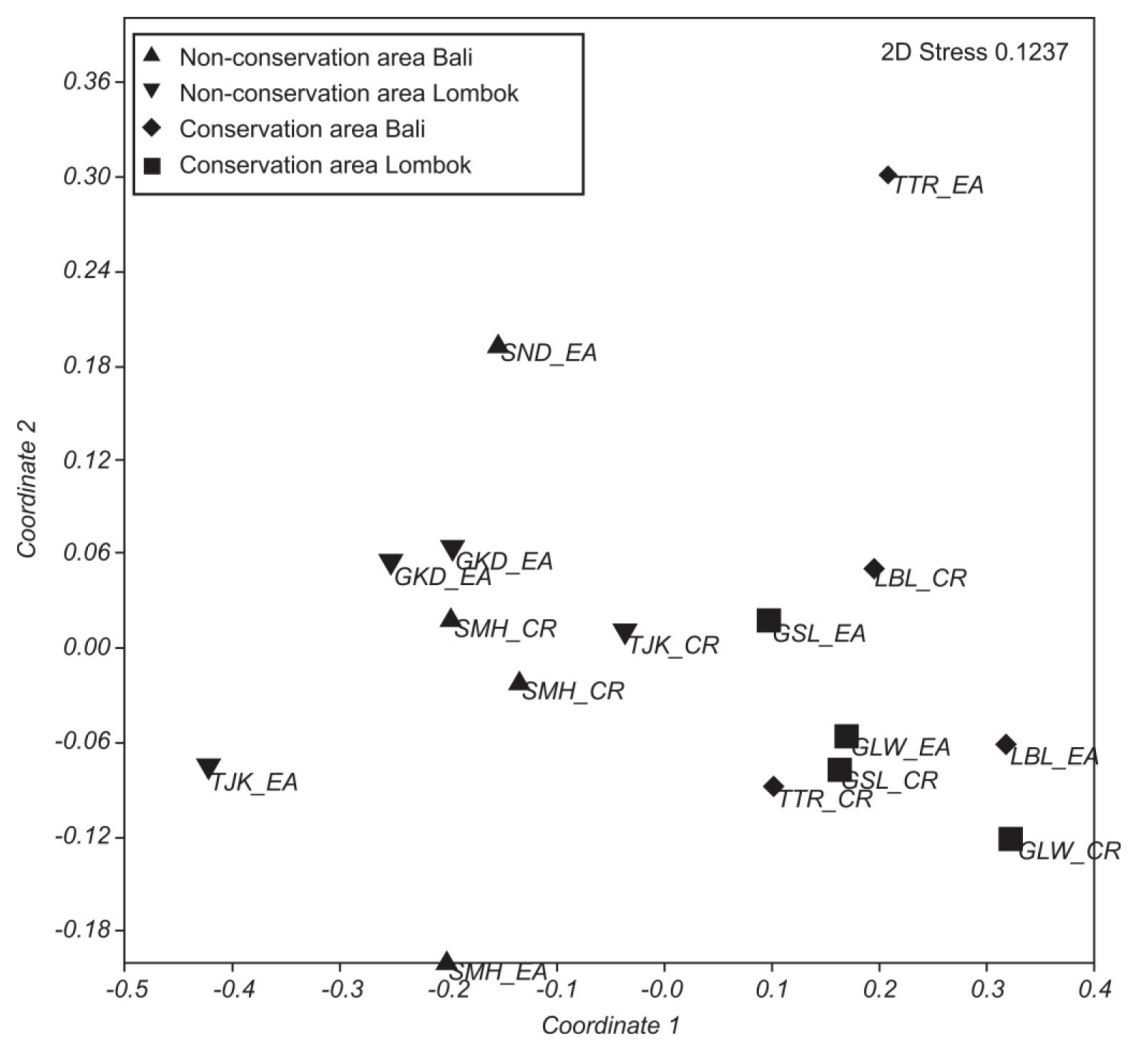
Two dimensional nMDS ordination based on abundance of microalgae epiphytes between group of non-conservation area and conservation area in Bali and Lombok. Ordination was based on Bray-Curtis similarity matrices constructed from untransformed percentage coverage data.

The ordination based on nMDS showed that the composition of microalgae epiphytic assemblages differed along NCA and CA in Bali and Lombok (Fig. 2). The low stress value of the nMDS (0.1237) indicates that the ordination was a good representation of the underlying dissimilarity values.

**Figure 3:**
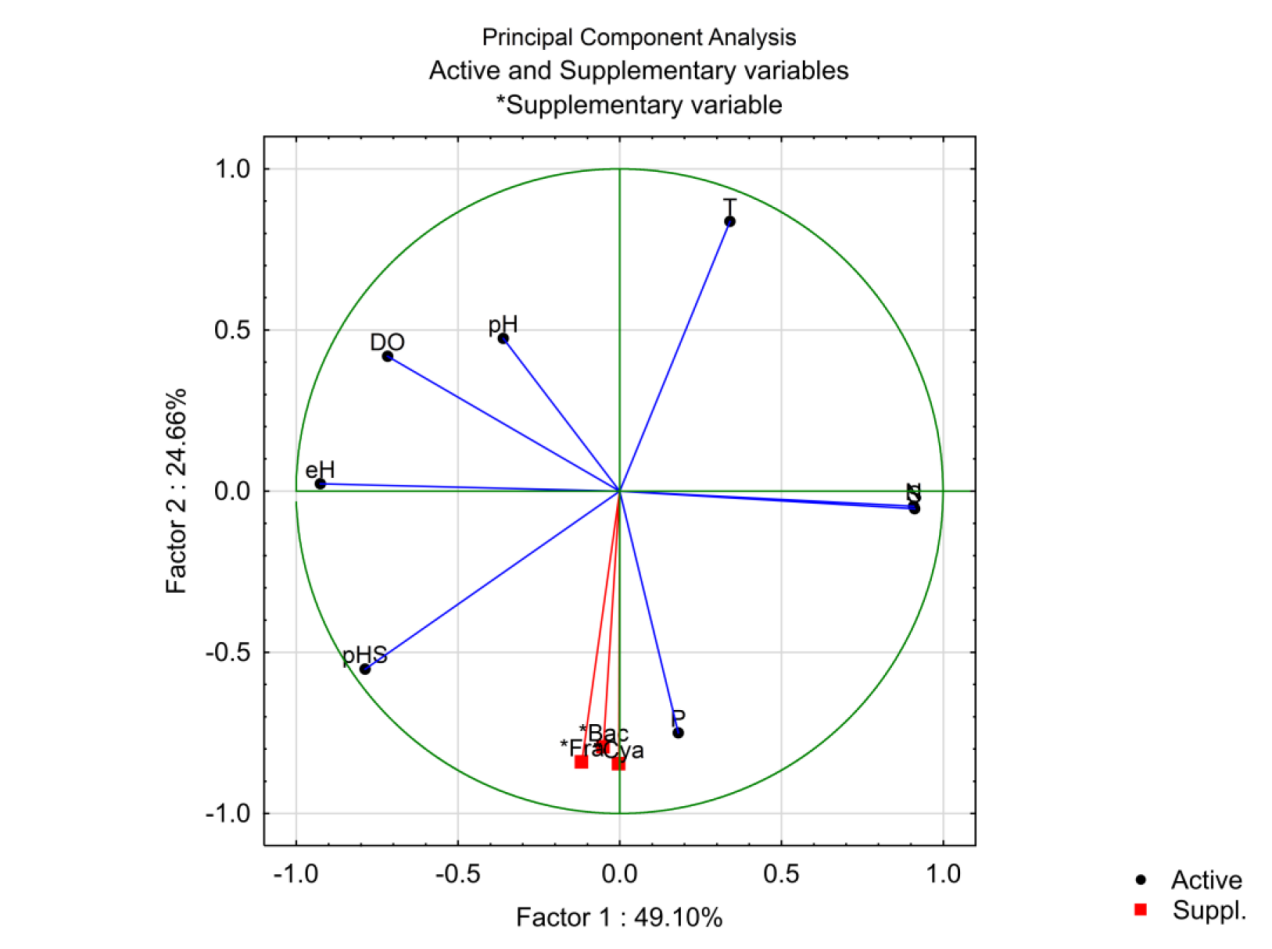
Principal component analysis (PCA) based on microalgae epiphytic abundance (supplementary variables) and physicochemical parameters (active variables) for all prospected sites on *E. acoroides* and *C. rotundata* leaves in Bali and Lombok.

The result of the PCA analysis showed that the first two axes accounted for 73.76% of the explained variability (Fig. 3). All the physicochemical parameters were separated into two groups. The first (factor 1) was consisted of dissolved oxygen (DO), redox potential (eH), pH in sediment (pHS), nitrate (N), and salinity (S). Meanwhile the second (factor 2) was consisted of pH in seawater (pH), temperature (T), phosphate (P), and all the supplementary variables which were the abundance of Bacillariophyceae (Bac), Cyanophyceae (Cya), and Fragillariophyceae (Fra) groups.

## DISCUSSION

### Comparison of Microalgae Epiphytic Communities Between Non-Conservation and Conservation Area

A clear differences of microalgae epiphytic assemblages between non-conservation area and conservation area were found in this study. The ordination of two dimensional (2D) nMDS shown on Fig. 2 in this study also confirmed a clear separation in distribution pattern of microalgae epiphytes assemblages between sites. The fundamental difference in the way assemblages of epiphytic algae are formed and maintained on each seagrass, hinting a complex interaction among environmental factors that influence the structure of epiphytes assemblages. There were some studies that shown the variety of environmental factors being responsible for controlling abundance, composition, and distribution pattern of microalgae epiphytes in their seagrass host (Lavery & Vanderklift 2002; Cornelisen & Thomas 2004; Fourqurean *et al*. 2010). Aside of environmental factors, the presence of macrograzers may influence the pattern and composition of microalgae epiphytes assemblages in seagrass meadows. Unfortunately, the presence-absence of macrograzers species were not taken in this study, yet previous study showed that grazing pressure become the most important variable in terms of variation epiphytic assemblages (Prado *et al*. 2007). Based on that fact, it is possible that some of these physicochemical parameters contribute to the differences of microalgae epiphytic assemblages between two different area in Bali and Lombok.

The studies about comparison of epiphytes assemblages between different meadows location have been widely carried out (Ugarelli *et al*. 2018; Balata *et al*. 2007). Besides of environmental factors and grazing pressure, conservation area which was relatively categorised as undisturbed location may provides more natural habitat to not only epiphytes species, but also its host seagrass to develop normally. Meanwhile, in the disturbed area tend to influence by highly anthropogenic pressure that directly impact on algae blooms and may lead to declines photosynthetic activity on seagrass (Orbita & Mukai 2013). Moreover, some of epiphytes like bryozoans, coarsely branched algae, and foraminifers communities were found significantly different between disturbed and undisturbed locations on *Posidonia oceanica* leaves at Tuscany (Italy), even there was no statistically evidence related to anthropogenic disturbance (Piazzi *et al*. 2004).

### Comparison of Microalgae Epiphytic Communities Between Seagrass Species

In this study, the differences of microalgae epiphytic assemblages between *E. acoroides* and *C. rotundata* leaves were found. The composition of microalgae epiphytic in *Enhalus acoroides* leaves was more diverse than those in *Cymodocea rotundata*. The abundance of microalgae epiphytic in *E. acoroides* leaves also higher than those in *C. rotundata*. These differences are likely to be related to morphological and longevity between the seagrasses. As shown in Fig. 3b, the LAI as the product of leaf surface between two seagrasses were strongly different each other. Due to their larger and longer leaf, *E. acoroides* provides more area for epiphytes to settle than in *C. rotundata*. The leaf of *E. acoroides* is ribbon-like, can be 200 cm long, and nearly 2 cm wide, while the leaf of *C. rotundata* is way much shorter, 7 cm–15 cm long, 0.2 cm–0.4 cm wide, linear and also flat (Shaffai 2011). Therefore, *E. acoroides* will gets advantages in terms of light availability and may explain the higher abundance and composition of epiphytes than those on *C. rotundata*. The relationship between the seagrass canopy by leaf shading and reduced the light availability confirmed in the previous studies (Enríquez & Pantoja-Reyes 2005; Lobelle *et al*. 2013).

Another aspect that plays important role in assemblages’ differences between those seagrasses is leaf growth. *C. rotundata* has the higher value of growth rates than *E. acoroides* (Brouns 1987; Rattanachot & Prathep 2011). So, in terms of slower growth rates, *E. acoroides* offer more time for microalgae epiphytic species to settle, colonise, mature and establish diverse communities. The same result found that the *Posidonia oceanica* which has slower growth rates than *C. nodosa*, has more diverse assemblages of epiphytes species (Mabrouk *et al*. 2014). Moreover, the evidence on microdistribution of cells on leaves showed that the density of cells tends to increased with the age of leaves on individual shoot of *Zostera* species, one of the seagrass which has rapid growth (Lebreton *et al*. 2009).

The leaf turnover of each seagrass may become another explanation about the differences of epiphytes composition and abundance. The relatively short turnover time of the *C. rotundata* leaves tend to have lower composition and abundance than those in *E. acoroides* which is more persistent seagrass. Table 4 showed that most epiphytes species on *C. rotundata* was dominates by *Oscillatoria sp*, which was identified as fast growing species (Prabakaran 2011). Meanwhile, *Enhalus acoroides* exhibits more diverse composition epiphytic species. This suggest that short-lived seagrass species tend to attach by rapid growing epiphytes species, while in long-lived seagrass species such as *E. acoroides* tend to have more dense, more mature and more diverse epiphytes because of long time recruitment. This point of view also confirmed on other studies which were compares microalgae assemblages and its spatial patterns on some different seagrass leaves (Whalen *et al*. 2013; Gartner *et al*. 2013; Uku *et al*. 2007; Balata *et al*. 2007). Another example showed that the high leaf turnover on *Zostera noltii* is linked with the absence of microalgae epiphytes closed to 0.001% of average biomass (Lebreton *et al*. 2009). This is implied that the short life span of seagrass leaves would thus prevent more epiphytes species to colonise, then would select specific species to attached as the pioneer.

### Influences of Physicochemical Parameters on Microalgae Epiphytic Communities

This study showed that the variation of seawater ad sediment physicochemical parameters strongly influenced the distribution and assemblages of microalgae epiphytes communities (Fig. 3). Three group of epiphytes communities observed in this study were Bacillariophyceae (diatoms), Cyanophyceae and Fragillariophyceae. Bacillariophyceae become the most frequently and dominant species, both on *E. acoroides* and *C. rotundata* leaves. There are two possible answer to this case. Firstly, the phenolic acid produced by seagrass become immune for Bacillariophyceae to able themselves attached on leaf’s surface and colonised first among other epiphytes species (Harrison & Durance 1985). Secondly, simply because of Bacillariophyceae have a smaller surface area and make them easier to attached. Many of them produce polysaccharides in thread form, mucus pads, or tubes that may enable to attach, despite currents and waxy cuticle (Corlett & Jones 2007).

The composition and abundance of Bacillariophyceae, Cyanophyceae and Fragillariophyceae strongly influenced by concentration of phosphate (P), pH in sediment (pHS) and temperature (T) (Fig. 3). The PCA analysis revealed that abundance of microalgae epiphytes has positive correlation with phosphate and pH in sediment, while for temperature showed negative correlation. These was implied that increasement of phosphate’s and pH concentration may lead to increase the abundance of microalgae epiphytes. Otherwise, higher temperature may lead the abundance of epiphytes declines, while when the temperature of seawater declined, the abundance increases.

Previous studies also state that phosphate is important features which promoted the growth of cyanobacteria and green algae (Joung *et al*. 2011). Another example of positive correlation between phosphate and seasonal variability of Chlorophyceae, Bacillariophyceae, Cyanophyceae and zooplankton density in Red River, Vietnam (Hoang *et al*. 2018). The relationship between water temperature and seasonally nutrient (nitrate and phosphate) to the seagrass and its component including epiphytes also found in Andaman Sea, Thailand (Wirachwong & Holmer 2010). The evidence about link between pH and epiphytes communities observed in *Zostera marina* species using electrochemical microsensors. The presence of epiphytes increase leaf surface pH up to 9.62 and makes plants starvation because of CO_2_ depletion (Brodersen *et al*. 2020).

Although many studies showed that environmental factors responsible for epiphytes communities (Wear *et al*. 1999; Piazzi *et al*. 2016; Lapointe *et al*. 2004; Campbell & Fourqurean 2014), the negative significant relationship between nutrient enrichment and epiphytes on seagrass reported in experimental assessment on *Thalassia testudinum* (Heck *et al*. 2000). This study observed small grazers were more influenced the structure and composition of microalgae epiphytes. Furthermore, grazing pressure (25%) reported as the most responsible and important variables that influenced variation of epiphytes assemblages, followed by nutrient availability (11%), meadow structure (6%), light (5%) and seagrass shoot length (4%) (Prado *et al*. 2007). A possible reason about different result with other studies is epiphytes responses to nutrient availability and environmental factors can be variable, very site-specific within region depend on seasonal variability and local competition with other species.

### Human Impact on Seagrass and Its Microalgae Epiphytic Communities

Human disturbances may alter the ecological role of seagrass and its microbiome including the laminates of microalgae epiphytes by possible additions of nutrients because of highly anthropogenic activities. Unfortunately, the temporal indicators of eutrophication (e.g., nutrient sources, turbidity, sedimentation and river discharge) were not taken in this study, yet previous study showed that seagrass ecosystems along with epiphytes are threatened by multiple human disturbances (Daby 2003; Karlina *et al*. 2018). Human activities such as disturbances of coastal land development, motorboating, some types of aquaculture, fishing practices likes trawling has led to increase the turbidity and act with algal overgrowth, from nutrient enrichment to promote seagrass die-off.

A generalised shift in biomass of seagrass, epiphytes, and microalgae was illustrated in shallow and deeper coastal marine (Burkholder *et al*. 2007). As nutrient enrichment increasing, lights became the primary limiting factors. This situation tends to proceed toward dominance of rapidly growing epiphytes and macroalgae, which are considered as competitors for light availability to seagrass. Same result also confirmed that eutrophication positively correlated to reduce above and below ground seagrass production, decrease shoot density, and increase the abundance of fast growing phytoplankton, benthic algae, and microalgae epiphytic (Coll *et al*. 2011; Houk & Camacho 2010). As the microalgae epiphytes rapidly grows, the oxygen production and respiration become increasingly uncoupled temporally, often responsible to hypoxic and anoxic conditions.

Another result has been observed on a pattern of spatial variability in the structure of epiphytes assemblages in relation to human interferences in Tunisia. The result showed that the diversity of epiphytes was reduced by the loss of the biomass and percentage cover near the source disturbed locations (Ben Brahim *et al*. 2010). As previously mentioned, increases in water turbidity in disturbed locations may become detrimental, either for seagrass or epiphytes since the light is restricted. Understanding interspecific differences within ecologically functional groups of microalgae epiphytic is critical to predict and potentially mitigate impacts of human-driven environmental changes, as ecosystem stability and resilience are enhanced by response diversity.

## CONCLUSION

This study revealed the significant differences about microalgae epiphytic communities between sites (non-conservation area – conservation area) and seagrass leaves (*E. acoroides – C. rotundata*). We proposed that these differences driven by complex interaction between environmental factors, morphology of seagrass leaves, longevity and shoot length. The implications of this microalgae epiphytic communities for the seagrass ecosystems is important, such as primary productivity, nutrient cycling, and directly influence the seagrass health. Since complex interactions between biotic factors, abiotic factors, seagrass as the host, and epiphytes communities’ interest to know, we recommend assessing not only seasonally but also spatially across wide range region for future studies. This is important to know to gets new insight and evaluate repercussions in the marine ecosystems as a whole.

## Figures and Tables

**Table 1:** Sites name, codes, categories and geographic coordinates included in our study.

Site	Code	Category	Latitude	Longitude
BALI				
Samuh	SMH	NCA	8°47′11.14″S	115°13′47.76″E
Shindu	SND	NCA	8°41′1.10″S	115°15′54.04″E
Teluk Terima	TTR	CA	8° 9′11.65″S	114°31′17.18″E
Labuhan Lalang	LBL	CA	8° 8′31.68″S	114°32′20.77″E
LOMBOK				
Tanjung Kelor	TJK	NCA	8°44′24.10″S	116° 1′32.10″E
Gili Kedis	GKD	NCA	8°43′50.66″S	116° 1′33.47″E
Gili Lawang	GLW	CA	8°17′39.74″S	116°42′27.62″E
Gili Sulat	GSL	CA	8°17′39.74″S	116°42′27.62″E

*Note*: NCA = non-conservation area; CA = conservation area.

**Table 2:** Physicochemical seawater and sediment taken in this study. All the parameters measured in situ and analysed in laboratory using follows methods.

Parameters	Units	Measurement device	Methods
Seawater			
Temperature	°C	pH pen meter	*In situ*
Salinity	‰	Refractometer	*In situ*
Dissolved oxygen	mg/L	DO meter	*In situ*
pH	-	pH pen meter	*In situ*
Nitrate	mg/L	Analysed in laboratory	Brucine
Phosphate	mg/L	Analysed in laboratory	Amm-Molybdat
Sediment			
pH	-	Soil pH meter	*In situ*
Redox potential (eH)	mV	Soil pH meter	*In situ*
Texture	-	Analysed in laboratory	Pipette

**Table 3:** Species richness of microalgae epiphytic species found on the leaves of *Enhalus acoroides* (EA) and *Cymodocea rotundata* (CR) in Bali.

Species name	SMH		SND		TTR		LBL	

	EA	CR	EA	CR	EA	CR	EA	CR
Bacillariophyceae								
*Amphora* sp.	13.47	18.20	33.33	15.20	41.47	42	18.93	8.93
*Coscinnodiscus* sp.	20.40	-	28.53	-	77.53	8.93	47.13	2.53
*Nitzchia* sp.	17.33	40.60	66.87	29.40	163	95.60	105.67	34.80
*Melosira* sp.	4.67	12.20	13.93	-	13.87	-	12.40	-
*Navicula* sp.	4.93	1.93	7.33	3.13	7.80	-	2.80	1
*Skeletonema* sp.	3.87	-	3.53	-	-	3.47	-	-
*Lycmophora* sp.	5.53	141.80	-	1.13	-	39.40	-	3.87
*Leptocylindrus* sp.	9.20	-	23.73	12.40	35.93	-	20.73	-
*Gyrosigma* sp.	-	-	-	-	0.53	1	2.20	-
*Biddulphia* sp.	-	-	0.87	-	-	-	-	-
*Fragillaria* sp.	0.87	1.80	-	-	3.60	-	2.93	2.40
*Surirella* sp.	-	4.47	-	-	2.20	-	4.67	-
*Thalassiothrix* sp.	-	8.87	-	-	10	-	10.07	-
*Pinnularia* sp.	8.47	-	-	9.40	18.47	-	7.93	-
*Thalassiosira* sp.	-	-	-	-	10.33	-	7.33	-
*Pseudonitzschia* sp.	-	-	12.93	-	1.13	-	-	-
*Cymbella* sp.	-	-	1.67	-	-	-	-	-
*Rhizosolenia* sp.	-	2.80	-	8	17.53	19.53	5.07	3.13
Cyanophyceae								
*Planktothrix* sp.	9.13	-	15.80	13.20	-	20.13	28.87	-
*Oscillatoria* sp.	14.93	62.13	49.33	33.87	94.20	80.47	145.93	31.20
*Lyngbya* sp.	-	25.60	9.33	-	42.67	8.40	20.47	-
Fragilariophyceae								
*Tabellaria* sp.	7.53	-	3.93	-	-	43.20	127.93	44.87

*Note*: SMH = Samuh; SND = Shindu; TTR = Teluk Terima; LBL = Labuhan Lalang.

**Table 4:** Species richness of microalgae epiphytic species found on the leaves of *Enhalus acoroides* (EA) and *Cymodocea rotundata* (CR) in Lombok.

Species name	TJK		GKD		GLW		GSL	

	EA	CR	EA	CR	EA	CR	EA	CR
Bacillariophyceae								
*Amphora* sp.	36.33	10.67	36.13	13.13	24.67	7	11	9.07
*Coscinnodiscus* sp.	-	-	17.07	-	6.47	-	2.33	-
*Nitzchia* sp.	61.80	14.27	140	55	108.40	9.47	17.93	17.73
*Melosira* sp.	-	-	-	-	-	-	-	-
*Navicula* sp.	11.47	-	30.80	15.93	7.27	2.13	9.20	2.27
*Skeletonema* sp.	-	-	-	-	8.60	-	-	-
*Lycmophora* sp.	4.67	-	2.80	2.47	-	-	-	-
*Leptocylindrus* sp.	-	8.13	22.20	14.40	11.27	-	13.20	-
*Gyrosigma* sp.	-	-	2.53	-	-	-	-	-
*Biddulphia* sp.	-	-	-	-	-	-	-	-
*Fragillaria* sp.	-	-	-	-	-	-	-	-
*Surirella* sp.	-	-	-	-	-	-	-	-
*Thalassiothrix* sp.	-	-	-	-	2.73	-	4.53	-
*Pinnularia* sp.	26.73	-	24.13	14.87	6.27	-	-	-
*Thalassiosira* sp.	-	-	-	-	-	-	-	-
*Pseudonitzschia* sp.	-	-	-	-	-	-	-	-
*Cymbella* sp.	-	-	-	-	-	-	-	-
*Rhizosolenia* sp.	11.53	-	8.13	17.73	13.27	8.73	11.20	6.53
Cyanophyceae								
*Planktothrix* sp.	-	-	18.93	-	19.87	-	7.13	-
*Oscillatoria* sp.	49.60	14.20	112.73	44.20	52.27	13.47	16.53	18.33
*Lyngbya* sp.	-	1.53	9.20	-	-	-	-	-
Fragilariophyceae								
*Tabellaria* sp.	7.20	-	2.80	11.87	-	-	-	-

*Note*: TJK = Tanjung Kelor; GKD = Gili Kedis; GLW = Gili Lawang; GSL = Gili Sulat.

**Table 5:** The result of analysis of similarity (ANOSIM) in microalgae epiphytes assemblages between sites and seagrass species at Bali and Lombok.

*Factor*	*Test Pairs*	*R value*	ρ
Between seagrass	EA Bali - CR Bali	0.3854	0.009*
	EA Lombok - CR Lombok	0.5209	0.008*
	EA Bali - EA Lombok	0.2501	0.003*
	CR Bali - CR Lombok	0.3627	0.001*
Between sites	NCA Bali - CA Bali	0.5625	0.0425**
	NCA Lombok - CA Lombok	0.3481	0.0089*
	NCA Bali - NCA Lombok	0.1533	0.027**
	CA Bali - CA Lombok	0.4603	0.002*

*Note*: Comparisons were made using Bray-Curtis similarity matrices based on untransformed percentage cover data with the number of permutations was 9999 in all cases.

* = *p* < 0.01;

** = *p* < 0.05.

**Table 6:** Community structure of microalgae epiphytes on the leaves of *E. acoroides* (EA) and *C. rotundata* (CR) in prospected sites at Bali and Lombok.

Sites and seagrass	Shannon-Wiener (H′)	Evenness (E)	Simpson (C)
NCA Bali	2.196	0.783	0.153
CA Bali	2.149	0.917	0.155
NCA Lombok	2.021	0.793	0.177
CA Lombok	1.710	0.843	0.221
EA Bali	2.326	0.963	0.130
CR Bali	2.019	0.737	0.178
EA Lombok	2.028	0.769	0.186
CR Lombok	1.703	0.867	0.213

**Table 7:** The result of similarity percentage (SIMPER) of microalgae epiphytes community (based on average dissimilarity 61.73% between sites and 53.1% between seagrass).

Taxon	Mean abundance	Average dissimilarity	% contribution	% cumulative
Between sites				
*Nitzchia* sp.	29	15.14	24.52	24.52
*Lycmophora* sp.	73.7	9.834	15.93	40.45
*Oscillatoria* sp.	38.5	7.999	12.96	53.41
*Coscinnodiscus* sp.	10.2	5.336	8.644	62.05
*Amphora* sp.	15.8	4.086	6.619	68.67
Between seagrass				
*Lycmophora* sp.	2.77	14.24	26.82	26.82
*Coscinnodiscus* sp.	24.5	6.233	11.74	38.56
*Oscillatoria* sp.	32.1	6.116	11.52	50.07
*Nitzchia* sp.	42.1	6.016	11.33	61.4
*Lyngbya* sp.	4.67	2.728	5.136	66.54

**Table 8:** Physicochemical and sediment parameters taken on each site at Bali and Lombok.

Parameters	SMH	SND	TTR	LBL	TJK	GKD	GLW	GSL
Seawater								
Temperature (°C)	29.38	31.21	26.73	27.81	33.2	34	29.3	29.2
Salinity (‰)	28.87	29	33	29.8	33.8	34	35.67	35.1
Dissolved oxygen (mg/L)	7.51	7.52	4.59	4.67	5.16	5.63	3.72	3.76
pH	7.92	7.67	7.55	7.53	7.49	7.79	7.76	7.7
Nitrate (mg/L)	0.09	0.08	0.37	0.11	0.63	0.34	0.22	0.59
Phosphate (mg/L)	0.23	0.16	0.83	0.1	0.26	0.27	0.2	0.2
Sediment								
pH	7.4	7.3	7.4	7.2	6.9	6.8	7.1	6.9
eH (mV)	104.8	110.2	74.4	110.1	48	73	74	77
Texture	Sand	Sand	Sandy clay	Sandy clay	Sand	Sand	Sand	Sand

## References

[b1-tlsr-32-2-97] Aho K, Beck E (2011). Effects of epiphyte cover on seagrass growth rates in two tidal zones. Dartmouth Undergraduate Journal of Science.

[b2-tlsr-32-2-97] Anderson MJ, Walsh DC (2013). PERMANOVA, ANOSIM, and the Mantel Test in the face of heterogeneous dispersions: What null hypothesis are you testing?. Ecological Monograph.

[b3-tlsr-32-2-97] Balata D, Nesti U, Piazzi L, Cinelli F (2007). Patterns of spatial variability of seagrass epiphytes in the north-west Mediterranean Sea. Marine Biology.

[b4-tlsr-32-2-97] Ben Brahim M, Hamza A, Hannachi I, Rebai A, Jarboui O, Bouain A, Aleya L (2010). Variability in the structure of epiphytic assemblages of *Posidonia Oceanica* in relation to human interferences in the Gulf of Gabes, Tunisia. Marine Environmental Research.

[b5-tlsr-32-2-97] Brodersen KE, Koren K, Revsbech NP, Kühl M (2020). Strong leaf surface basification and CO2 limitation of seagrass induced by epiphytic biofilm microenvironments. Plant Cell and Environment.

[b6-tlsr-32-2-97] Brouns JWM (1987). Growth patterns in some indo-west-pacific seagrasses. Aquatic Botany.

[b7-tlsr-32-2-97] Burkholder JM, Tomasko DA, Touchette BW (2007). Seagrasses and eutrophication. Journal of Experimental Marine Biology and Ecology.

[b8-tlsr-32-2-97] Campbell JE, Fourqurean JW (2014). Ocean acidification outweighs nutrient effects in structuring seagrass epiphyte communities. Journal of Ecology.

[b9-tlsr-32-2-97] Cheng CC (2004). Statistical approaches on discriminating spatial variation of species diversity. Botanical Bulletin of Academia Sinica.

[b10-tlsr-32-2-97] Chung MH, Lee KS (2008). Species composition of the epiphytic diatoms on the leaf tissues of three Zostera species distributed on the southern coast of Korea. Algae.

[b11-tlsr-32-2-97] Clarke KR (1993). Non-parametric multivariate analyses of changes in community structure. Australian Journal of Ecology.

[b12-tlsr-32-2-97] Coll M, Schmidt A, Romanuk T, Lotze HK (2011). Food-web structure of seagrass communities across different spatial scales and human impacts. PLoS ONE.

[b13-tlsr-32-2-97] Corlett H, Jones B (2007). Epiphyte communities on *Thalassia testudinum* from Grand Cayman, British West Indies: Their composition, structure, and contribution to lagoonal sediments. Sedimentary Geology.

[b14-tlsr-32-2-97] Cornelisen CD, Thomas FIM (2004). Ammonium and nitrate uptake by leaves of the seagrass *Thalassia testudinum*: Impact of hydrodynamic regime and epiphyte cover on uptake rates. Journal of Marine Systems.

[b15-tlsr-32-2-97] Crump BC, Koch EW (2008). Attached bacterial populations shared by four species of aquatic angiosperms. Applied and Environmental Microbiology.

[b16-tlsr-32-2-97] Daby D (2003). Effects of seagrass bed removal for tourism purposes in a Mauritian Bay. Environmental Pollution.

[b17-tlsr-32-2-97] Duarte CM (2000). Marine biodiversity and ecosystem services: An elusive link. Journal of Experimental Marine Biology and Ecology.

[b18-tlsr-32-2-97] Enríquez S, Pantoja-Reyes NI (2005). Form-function analysis of the effect of canopy morphology on leaf self-shading in the seagrass *Thalassia testudinum*. Oecologia.

[b19-tlsr-32-2-97] Fourqurean JW, Muth MF, Boyer JN (2010). Epiphyte loads on seagrasses and microphytobenthos abundance are not reliable indicators of nutrient availability in oligotrophic coastal ecosystems. Marine Pollution Bulletin.

[b20-tlsr-32-2-97] Gartner A, Tuya F, Lavery PS, McMahon K (2013). Habitat preferences of macroinvertebrate fauna among seagrasses with varying structural forms. Journal of Experimental Marine Biology and Ecology.

[b21-tlsr-32-2-97] Güreşen A, Güreşen SO, Aktan Y (2020). Temporal and bathymetric variation of epiphytic microalgae on *Posidonia oceanica* (L.) delile leaves in Gökçeada (North Aegean, Turkey). Turkish Journal of Fisheries and Aquatic Sciences.

[b22-tlsr-32-2-97] Hamisi M, Díez B, Lyimo T, Ininbergs K, Bergman B (2013). Epiphytic cyanobacteria of the seagrass *Cymodocea rotundata*: Diversity, diel NifH expression and nitrogenase activity. Environmental Microbiology Reports.

[b23-tlsr-32-2-97] Harrison PG, Durance CD (1985). Reductions in photosynthetic carbon uptake in epiphytic diatoms by water-soluble extracts of leaves of *Zostera marina*. Marine Biology.

[b24-tlsr-32-2-97] Heck KL, Pennock J, Valentine JF, Coen LD, Sklenar SA (2000). Effects of nutrient enrichment and small predator density on seagrass ecosystems: An experimental assessment. Limnology and Oceanography.

[b25-tlsr-32-2-97] Hoang HTT, Duong TT, Nguyen KT, Le QTP, Luu MTN, Trinh DA, Le AH (2018). Impact of anthropogenic activities on water quality and plankton communities in the day river (Red River Delta, Vietnam). Environmental Monitoring and Assessment.

[b26-tlsr-32-2-97] Hoppenrath M, Elbrachter M, Drebes G (2009). Marine phytoplankton: Selected microphytoplankton species from the North Sea around Helgoland and Sylt, Kleine Senckenberg-Reihe 49.

[b27-tlsr-32-2-97] Houk P, Camacho R (2010). Dynamics of seagrass and macroalgal assemblages in Saipan Lagoon, Western Pacific Ocean: Disturbances, pollution, and seasonal cycles. Botanica Marina.

[b28-tlsr-32-2-97] Joung SH, Oh HM, Ko SR, Ahn CY (2011). Correlations between environmental factors and toxic and non-toxic microcystis dynamics during bloom in Daechung Reservoir, Korea. Harmful Algae.

[b29-tlsr-32-2-97] Karlina I, Kurniawan F, Idris F (2018). Pressures and status of seagrass ecosystem in the coastal areas of North Bintan, Indonesia. E3S Web of Conferences.

[b30-tlsr-32-2-97] Kocak F, Aydin-Onen S (2014). Epiphytic bryozoan community of *Posidonia oceanica* (L.) delile leaves in two different meadows at disturbed and control locations. Mediterranean Marine Science.

[b31-tlsr-32-2-97] Lapointe BE, Barile PJ, Matzie WR (2004). Anthropogenic nutrient enrichment of seagrass and coral reef communities in the lower Florida keys: Discrimination of local versus regional nitrogen sources. Journal of Experimental Marine Biology and Ecology.

[b32-tlsr-32-2-97] Larink O, Westheide W (2006). Coastal plankton: Photo guide for European Sea.

[b33-tlsr-32-2-97] Lavery PS, Vanderklift MA (2002). A comparison of spatial and temporal patterns in epiphytic macroalgal assemblages of the seagrasses *Amphibolis griffithii* and *Posidonia coriacea*. Marine Ecology Progress Series.

[b34-tlsr-32-2-97] Lavery PS, Reid T, Hyndes GA, Van Elven BR (2007). Effect of leaf movement on epiphytic algal biomass of seagrass leaves. Marine Ecology Progress Series.

[b35-tlsr-32-2-97] Lebreton B, Richard P, Radenac G, Bordes M, Bréret M, Arnaud C, Mornet F, Blanchard GF (2009). Are epiphytes a significant component of intertidal *Zostera noltii* beds?. Aquatic Botany.

[b36-tlsr-32-2-97] Leliaert F, Van reusel W, Clerck ODE, Coppejans E (2001). Epiphytes on the seagrasses of Zanzibar Island (TANZANIA), floristic and ecological aspects. Belgian Journal of Botany.

[b37-tlsr-32-2-97] Lobelle D, Kenyon EJ, Cook KJ, Bull JC (2013). Local competition and metapopulation processes drive long-term seagrass-epiphyte population dynamics. PLoS ONE.

[b38-tlsr-32-2-97] Mabrouk L, Brahim MB, Hamza A, Mahfoudhi M, Bradai MN (2014). A comparison of abundance and diversity of epiphytic microalgal assemblages on the leaves of the seagrasses *Posidonia oceanica* (L.) and *Cymodocea nodosa* (Ucria) asch in Eastern Tunisia. Journal of Marine Biology.

[b39-tlsr-32-2-97] Marco-Méndez C, Ferrero-Vicente LM, Prado P, Heck KL, Cebrián J, Sánchez-Lizaso JL (2015). Epiphyte presence and seagrass species identity influence rates of herbivory in Mediterranean seagrass meadows. Estuarine, Coastal and Shelf Science.

[b40-tlsr-32-2-97] Medlin LK, Juggins S (2018). Multivariate analyses document host specificity, differences in the diatom metaphyton vs. epiphyton, and seasonality that structure the epiphytic diatom community. Estuarine, Coastal and Shelf Science.

[b41-tlsr-32-2-97] Nelson WG (2017). Development of an epiphyte indicator of nutrient enrichment: Threshold values for seagrass epiphyte load. Ecological Indicators.

[b42-tlsr-32-2-97] Odum EO (1971). Fundamental of Ecology.

[b43-tlsr-32-2-97] Olds AD, Connolly RM, Pitt KA, Pittman SJ, Maxwell PS, Huijbers CM, Moore BR (2016). Quantifying the conservation value of seascape connectivity: A global synthesis. Global Ecology and Biogeography.

[b44-tlsr-32-2-97] Orbita MLS, Mukai H (2013). Relationship between epiphytes and the photosynthetic activity of temperate seagrasses. Advances in Agriculture & Botanics-International Journal of the Bioflux Society [AAB Bioflux].

[b45-tlsr-32-2-97] Pardi G, Piazzi L, Balata D, Papi I, Cinelli F, Benedetti-Cecchi L (2006). Spatial variability of *Posidonia oceanica* (L.) delile epiphytes around the mainland and the islands of Sicily (Mediterranean Sea). Marine Ecology.

[b46-tlsr-32-2-97] Piazzi L, Balata D, Ceccherelli G (2016). Epiphyte assemblages of the Mediterranean seagrass *Posidonia oceanica*: An overview. Marine Ecology.

[b47-tlsr-32-2-97] Piazzi L, Balata D, Cinelli F, Benedetti-Cecchi L (2004). Patterns of spatial variability in epiphytes of *Posidonia oceanica*: Differences between a disturbed and two reference locations. Aquatic Botany.

[b48-tlsr-32-2-97] Prabakaran M (2011). Invitro antimicrobial potentials of Marine Oscillatoria species. Asian Journal of Plant Science and Research.

[b49-tlsr-32-2-97] Prado P, Alcoverro T, Martínez-Crego B, Vergés A, Pérez M, Romero J (2007). Macrograzers strongly influence patterns of epiphytic assemblages in seagrass meadows. Journal of Experimental Marine Biology and Ecology.

[b50-tlsr-32-2-97] Rattanachot E, Prathep A (2011). Temporal variation in growth and reproduction of *Enhalus acoroides* (L.f.) royle in a monospecific meadow in Haad Chao Mai National Park, Trang Province, Thailand. Botanica Marina.

[b51-tlsr-32-2-97] Reyes J, Sanson M (2001). Biomass and production of the epiphytes on the leaves of *Cymodocea nodosa* in the Canary Islands. Botanica Marina.

[b52-tlsr-32-2-97] Shaffai AE (2011). Seagrasses of the Red Sea.

[b53-tlsr-32-2-97] Short FT, Coles RG (2001). Global seagrass research method.

[b54-tlsr-32-2-97] Ugarelli K, Laas P, Stingl U (2018). The microbial communities of leaves and roots associated with turtle grass (*Thalassia testudinum*) and manatee grass (*Syringodium filliforme*) are distinct from seawater and sediment communities, but are similar between species and sampling sites. Microorganisms.

[b55-tlsr-32-2-97] Uku J, Björk M, Bergman B, Díez B (2007). Characterization and comparison of prokaryotic epiphytes associated with three East African seagrasses. Journal of Phycology.

[b56-tlsr-32-2-97] Wear DJ, Sullivan MJ, Moore AD, Millie DF (1999). Effects of water-column enrichment on the production dynamics of three seagrass species and their epiphytic algae. Marine Ecology Progress Series.

[b57-tlsr-32-2-97] Whalen MA, Duffy JE, Grace JB (2013). Temporal shifts in top-down vs. bottom-up control of epiphytic algae in a seagrass ecosystem. Ecology.

[b58-tlsr-32-2-97] Wirachwong P, Holmer M (2010). Nutrient dynamics in 3 morphological different tropical seagrasses and their sediments. Aquatic Botany.

